# Exploring subjective constructions of health in China: a Q-methodological investigation

**DOI:** 10.1186/s12955-020-01414-z

**Published:** 2020-06-03

**Authors:** Zhuxin Mao, Shenaz Ahmed, Christopher Graham, Paul Kind

**Affiliations:** 1 Leeds Institute of Health Sciences, University of Leeds, Leeds, UK; 2 Department of Psychology, Queen’s University Belfast, Belfast, UK

**Keywords:** Health-related quality of life, Health, Cultural differences, China, Q-methodology, Qualitative study

## Abstract

**Background:**

With an increasing awareness of people’s satisfaction and feeling, health-related quality of life (HRQoL) has become an essential aspect of measuring health. HRQoL is fundamentally a foreign concept introduced to China from the West. While a growing number of studies applied western HRQoL measures, few content validity tests examined the legitimacy of applying Western developed HRQoL measures in a Chinese cultural setting. If there are distinct differences in health conceptualisation between China and the West, it can be argued that those western measures may fail to ask the most appropriate and important questions among a Chinese population in assessing health. As a limited number of studies have investigated Chinese people’s understandings of health, this study aimed to explore how health is defined and described in China.

**Methods:**

A Q-methodological study was conducted to explore subjective constructions of health among Chinese participants. A scoping review of Chinese generic HRQoL measures, supplemented by a series of qualitative interviews conducted in China, produced a list of 42 statements representing aspects of health considered as being important in a Chinese cultural setting. Chinese participants in face-to-face interviews ranked and sorted these statements. Data were analysed to identify clusters of participants who shared a similar perspective, using a by-person factor analysis procedure.

**Results:**

110 Chinese participants with various demographics characteristics completed sorting interviews. Five independent factors emerged: (I) “Physical independence and social interaction skills”; (II) “Physical health”; (III) “Sensations and feelings”; (IV) “Lifestyles”; (V) “Learning and working abilities”.

**Conclusions:**

The Q-study showed that many health statements were rated highly as most important by a diverse range of Chinese participants but were not covered in the commonly used Western HRQoL measure EQ-5D. It then suggests that the EQ-5D descriptive system might need modification to improve its capacity to measure health status in China. The study thus raises a general question as to how appropriate the Western-developed HRQoL measures are when used to assess health in a significantly different cultural setting.

## Introduction

With an increasing awareness of people’s satisfaction and feeling, health-related quality of life (HRQoL) has become an essential aspect of measuring health [1, 2]. Most of the commonly used HRQoL questionnaires have been developed in Europe or North America, with their descriptive systems being subsequently translated into other languages to be used worldwide. Although a growing number of studies use western HRQoL measures, few studies have considered cultural differences in conceptual equivalence [3–5], while those assessing cross-cultural equivalence normally focus on statistical psychometric properties [5].

Taking the use of EQ-5D in China as an example, the Chinese versions of EQ-5D have been widely used in China, including general population and patient-specific studies [6, 7]. The high ceiling effect is one of the problems encountered when using EQ-5D in China [8–11], suggesting that it may be inefficient to identify differences in health status for much of the Chinese population [12]. The proportion of people reporting a ceiling effect of EQ-5D in China (87% in the national population study in the year 2008) was much greater than European countries like UK, Sweden, and Germany where the proportions reporting no problem were 45, 42, and 66%, respectively. It may be because Chinese people are generally healthier than people living in the West, but this explanation is contradicted by data showing poorer life expectancy, mortality or morbidity in China. A more reasonable hypothesis is that the cultural differences between China and the West make the European-developed questionnaire less effective. The questionnaire was also found to be less sensitive in detecting differences in health status [13, 14]. Additionally, its test-retest reliability is questioned by Chinese researchers [15].

Although various validation studies for the Chinese version of EQ-5D have been conducted, they focused on statistical tests, examining psychometric properties such as construct validity, reliability and responsiveness, while few studies addressed conceptual equivalence issues [16–19]. Given potential differences in how health is conceptualised in China and the West [20–23], it can be argued that EQ-5D may fail to ask the most appropriate and important questions for Chinese populations in assessing health.

Chinese papers on health concepts are predominantly theoretical and rarely collect data with members of the general population [24, 25]. This study thus investigated Chinese participants’ subjective understandings of key concepts that should be used to judge health. By comparing lay Chinese people’s understandings of health with a commonly-used Western HRQoL measure (EQ-5D), this study aimed to explore cultural differences in defining and measuring health between China and the West.

## Methods

### Design

The study was reviewed and approved by the School of Medicine Research Ethics Committee at the University of Leeds (reference number: MREC17–021).

A Q methodological study was used to fulfil the study objective. Q methodology was introduced by William Stephenson in 1935 as a way to scientifically assess subjective viewpoints [26, 27]. It is an effective approach for combining both qualitative and quantitative techniques to observe individuals’ personal opinions and identify patterns of views across a participant group [28, 29]. It has been used in various health-related studies to investigate concepts of QoL, experiences of pain and understandings of illnesses [30–32]. It has been used in Chinese populations in different research areas such as education, tourism, nursing and political science [33–36]. Q methodology comprises several steps: concourse development (wide collection of statements based on things written/said on the research topic – via scoping review and qualitative interviews), Q-sample generation (selection of statements from the concourse to enable participants to express different viewpoints), Q-sorting administration (participants rank statements), factor analysis and interpretation [26]. These steps are explained in detail in the following sections.

### Developing concourse and devising the Q-sample

The first step of a Q-study is to develop the “concourse”, which ‘consists of the things that are written or said about a topic that can be ‘socially contested, argued about and debated … matters of values and beliefs’ [37]. The development of the concourse of this study involved: (i) a scoping review of Chinese generic HRQoL measures, and (ii) qualitative interviews conducted in China focusing on aspects of health considered important in judging health for a Chinese population. Referring to the methodological framework of scoping review [38], currently available HRQoL measures that were developed in a Chinese cultural setting were identified. Attributes that were covered by those HRQoL measures and could be used in subjective health assessment were systematically summarised to develop a Chinese conceptual framework of health. Subsequently, a series of semi-structured face-to-face interviews were conducted to ask participants to talk about health. They were asked to describe their own health as well as to illustrate someone in good/poor health. This explored how Chinese lay people describe and appraise health to justify the conceptual framework and to identify any additional health concepts. The resulting conceptual framework included a wide range of health attributes likely to be considered important by a Chinese population.

Based on the conceptual framework, the Q-sample was generated. The Q-sample is a set of statements that include the diversity of opinions and perspectives about the research topic so that participants may rank statements to express their views [26]. The five dimensions of EQ-5D were also generated as statements, as a way to compare the descriptive system with other “Chinese-specific” statements. A draft version of Q-sample on various health-related aspects of subjective experiences, feelings or perceptions was generated. A more detailed process of how the conceptual framework was transformed into the Q-sample is presented in the Additional file 1. The ‘condition of instruction’ (guide for participants to sort the Q-sample) was “When judging a person’s health, how important is it to know about their ___?”

The draft Q-sample was sent to 10 Chinese people (two Chinese clinicians, two Chinese academic researchers who had worked on HRQoL projects and six lay people) for comments. They were asked to identify those unclear statements, after which they were asked to give reasons why they thought these statements unclear and/or suggest alternative wording. They were also asked to indicate if there were any similar statements. As a result of feedback from participants, the statements were then revised to eliminate ambiguity and repetition and ensured readability to lay people. Five pilot Q-sorts were subsequently conducted. As the participants of the pilot study confirmed that they understood the statements and had no problem in following instructions, no further revisions were made on the Q-sample. The final version of the Q-sample contained 42 statements.

### Participants

To explore the diversity of views, a group of Chinese participants (with Chinese nationality; living in China; using Chinese as the mother tongue;18 years old or older) with various demographic characteristics, including age, gender, geographical locations, rural/urban areas, educational background and his/her health condition, were purposively recruited. As the study required participants to comprehend, compare and rank 42 statements written in Chinese, participants were expected to be able to read and communicate in Mandarin. Potential participants were not recruited if they had cognitive problems or had a serious health condition that may limit their ability to complete the Q-sorting exercise.

Participants were identified and recruited through various social groups (such as a Mahjong game club, a nursing home and a village community), where group organisers were contacted to help the researcher to target and access potential participants. For example, a member of a Mahjong game club agreed to ask other members (mostly people in middle or elder age) if they were interested in participating in this study; a manager of a nursing home helped to contact people who had long-term health problems; a village head offered help in distributing recruitment leaflets and introducing the researcher to his villagers. The snowballing approach was also used by asking interviewees to suggest potential participants. Once prospective interviewees had confirmed their willingness to participate, the place, date and time were discussed and arranged. For the privacy of interviewees and the quality of interviews, interviewing places were selected carefully to ensure interviews could take place with minimal interruption. The chosen sites were various, including private meeting rooms in public teahouses, quiet compartments in cafes, meeting rooms in the places of interviewees’ employment. In the end, 110 participants from cities and villages in Southwest China (Chongqing), East China (Shanghai, Jiangsu, Zhejiang) and North China (Beijing and Tianjin), completed the Q-sort exercise. See Table 1 for sample characteristics.

**Table 1:** Demographic characteristics of participants (*n* = 110)

		Number (percentage)
Gender	Male	57 (52%)
	Female	53 (48%)
Age	< 40	44 (40%)
	40–60	35 (32%)
	60+	31 (28%)
Education background	Under high school	20 (18%)
	High school	14 (13%)
	Secondary specialised	15 (14%)
	College	18 (16%)
	University	42 (38%)
Self-rating health status using EQ-5D^a^	11111	42 (38%)
	11112	15 (14%)
	11121	16 (15%)
	11122	14 (13%)
	Other	22 (20%)
Self-rating health score	80–100	69 (63%)
	60–80	35 (32%)
	< 60	5 (5%)
Residence place	City	63 (57%)
	Non-city	47 (43%)
Region	Southwest China	54 (49%)
	East China	34 (31%)
	North China	13 (12%)
	Other	9 (8%)

^a^The Chinese version of the EQ-5D-5 L questionnaire was provided to each participant to complete after the sorting exercise. One participant declared he did not have time for completing the questionnaire and his health status information was missing

### Q-sorting

**Fig. 1:**
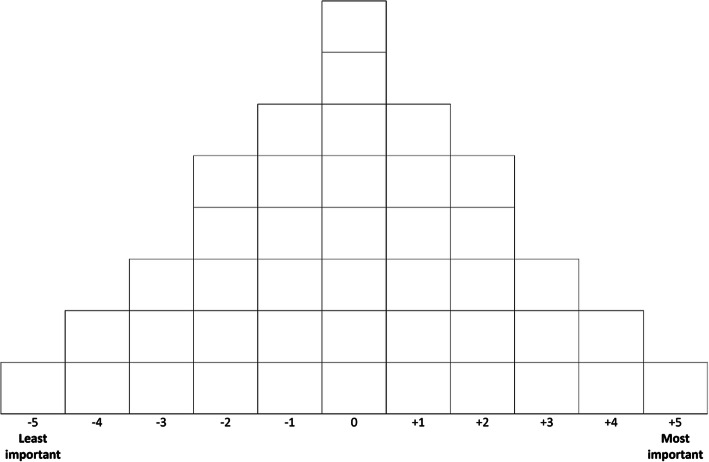
Q-Grid

Participants were provided with the Q-sample (42 statements individually printed on numbered cards) and a Q-grid (See Fig. 1). The sorting exercise was conducted individually by participants. Participants were asked to read each statement carefully and split them into three piles: “a pile for statements that you think are most important”; “a pile for statements that you think are least important” and “a pile for the rest”. Participants were then asked to sort the cards onto the Q-grid from most important (+ 5) to least important (− 5). For example, participants needed to place one statement that was most important to him/her on the rightmost blank cell and two second most important statements on the (+ 4) column and so on, until all the statements were assigned on the grid.

Participants were asked to check their completed Q-sort (distribution of statements on the Q-grid) and make any changes. The researcher took a photograph of the completed Q-sorts. Finally, the researcher conducted post-sorting interviews to explore why participants ranked statements as they did. Examples of post-sorting questions are presented in the Additional file 1. These interviews were audio-recorded.

### Factor analysis and rotation

**Fig. 2:**
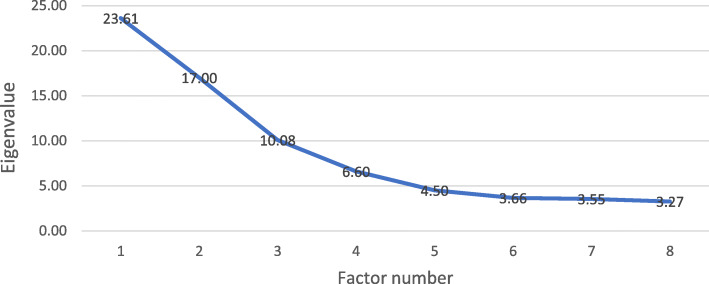
Scree Test

Participants’ Q-sorts were entered into PQMethod version 2.35 (available on http://schmolck.org/qmethod/) for analysis. The study adopted Principal Component Analysis with Varimax rotation to analyse the data, where similarities within factors and differences across them are maximised [39]. The next step was to determine how many factors should be retained for rotation and interpretation. The principal aim of factor extraction was to keep those factors that were reasonably interpretable and represented a distinct viewpoint [29]. The commonly adopted standards include selecting factors with eigenvalues greater than 1.00 [26, 29] and on which the Q-sorts of at least two exemplars load significantly [26, 40]. The eigenvalue (characteristics value) of a factor is closely associated with the variance accounted for by that factor (Eigenvalue = the variance accounted for by that factor × number of participants/100) [26]. Additionally, the Scree test has also been applied in many studies [41], where eigenvalues would be plotted on a line chart. The slope of the line would indicate which factors should be retained: those factors to the left of the point where the slope is evidently levelling off. The graph below (Fig. 2) draws the scree plot with the eigenvalues generated for each factor in this study. The graph shows a five-factor solution was potentially eligible for interpretation. The five factors explained 55% of the study variance and appeared to represent distinct viewpoints.

### Interpretation

For each factor, exemplars were identified (participants with Q-sorts loading + 0.4 (*p* < 0.01) on one factor only). These exemplars were merged in PQMethod to produce a factor array, a single ‘ideal’ Q-sort that best represented each factor (Table 2). The factor array for each factor represents how a participant with a correlation coefficient of 1 would have ranked the 42 statements. The pattern of ranked statements within each factor array was interpreted to identify the different viewpoints within the study sample [26]. Interpretation of each factor can be achieved by observing the scores of Q-sample in its factor array [37] as well as by recognising similarities and differences across the factors [40]. A lower value indicated that the statement was less important (for example, ‘-5’ suggested that the statement was least important) while a higher value indicated that the statement was more important. This interpretation process was supplemented by exemplars’ comments collected during the post-sorting interviews. The audio recordings of their comments were transcribed. The rationale of their sort, which can be referred to by reading the transcripts, helped to verify the initial interpretations of each factor.

**Table 2:** Factor Arrays: scores against each item by factor

Statements	Factor Arrays				
	1	2	3	4	5
1. Body constitution that can indicate the susceptibility to diseases	2	3	5	3	3
2. Ability to adapt to weather changes	−3	−1	−3	− 1	−5
3. Body weight	−2	0	−1	0	−1
4. Spiritual appearance	2	2	4	3	0
5. Natural colour and appearance of face	−2	1	−2	1	−4
6. Feeling of tiredness	−1	1	1	−1	−2
7. Body strength of doing things	−1	2	0	−1	2
8. Feeling of discomfort	−2	2	2	0	−2
9. Feeling of pain	−1	4	2	2	−3
10. Desire of having food	0	2	0	3	−3
11. Feeling of pressure	−1	0	2	−3	3
12. Feeling of depression	−2	1	3	−3	0
13. Feeling of anxiety	−1	0	2	−2	1
14. Tendency of being angry	−2	−1	0	−1	− 1
15. Feeling of fear	−5	−2	− 2	−5	0
16. Feeling of loneliness	−3	−2	−2	−4	−1
17. Self-confidence	3	−2	1	2	2
18. Ability to remain stable and peaceful in mood	2	0	1	0	1
19. Sleep quality	1	3	3	4	0
20. Ability to walk about	3	4	−1	−4	2
21. Ability to perform usual activities	4	3	0	−2	−1
22. Vision	0	1	−5	0	5
23. Hearing	0	0	−4	1	4
24. Ability to communicate with people	1	−2	−1	1	0
25. Ability to wash and dress oneself	5	5	0	−3	0
26. Dependence on medication	−3	1	0	−2	−2
27. State of sex life	−4	−2	−4	− 2	0
28. Ability to think things clearly	2	0	−1	0	2
29. Ability to perceive changes in surrounding and to respond swiftly	0	0	−2	−1	1
30. Ability to remember things	1	−1	−3	2	1
31. Ability to make decisions	−1	−4	−3	1	0
32. Ability to concentrate	0	−1	−2	2	2
33. State of social relations	1	−4	0	1	− 2
34. Ability to adapt to the social environment	3	−3	0	−1	− 1
35. Support from one’s social network	0	−3	−1	−2	−2
36. Social morality	1	−5	−1	0	1
37. Life attitude	4	−1	4	2	4
38. “Breadth of mind”	2	−3	1	1	3
39. Regularity in daily life	0	1	3	4	1
40. Diet habits	0	0	2	5	−4
41. Sense of satisfaction with life	1	−1	1	0	−3
42. Family medical history	−4	2	1	0	−1

## Results

Demographic information about exemplars in each factor is presented in Table 3.

**Table 3:** Demographic characteristics for exemplars in each factor

		Factor 1 (*n* = 19)	Factor 2 (*n* = 25)	Factor 3 (*n* = 16)	Factor 4 (*n* = 6)	Factor 5 (*n* = 4)
Gender	Male	7	8	10	6	3
	Female	12	17	6	0	1
Age	< 40	4	13	12	1	3
	40–60	7	4	3	3	1
	60+	8	8	1	2	0
	(Mean age)	55	43	34	52	30
Education background	Under high school	3	6	3	1	0
	High school	4	1	2	1	0
	Secondary	3	1	1	3	1
	College	3	4	2	1	1
	University	6	13	8	0	2
Self-rating health state using EQ-5D	11111	8	7	8	4	0
	11112	2	3	2	0	0
	11121	3	5	1	0	1
	11122	1	3	2	2	1
	Other	5	6	3	0	2
Self-rating health score	80–100	10	17	9	3	3
	60–80	8	4	7	3	1
	< 60	1	3	0	0	0
Residence place	City	12	13	10	2	3
	Non-city	7	12	6	4	1
Region	Southwest China	13	12	4	3	1
	East China	5	4	7	3	2
	North China	0	5	4	0	1
	Other	1	4	1	0	0

### Factor one: physical independence and social interaction skills

Q-sorts of 19 participants exemplified this factor.

Factor One exemplars tended to agree that one’s physical function was important in judging his/her health. Statements including “Ability to wash and dress oneself” (+ 5), “Ability to perform usual activities (such as working, studying, shopping, doing housework)” (+ 4) and “Ability to walk about” (+ 3) were ranked as most important. Participants revealed that it was essential to be physically independent and keep self-control over one’s own life, and according to them, being able to take care of themselves, to conduct usual activities and to walk around were all basic requirements in obtaining such independence. Exemplars who lost or partly lost physical independence explained that their daily activities had been significantly restricted, therefore their quality of life was largely damaged. Those participants who were physically well held the same point that one’s physical function was a fundamental component in life because they believed that people could have other pursuits, such as happiness and fortunes, only if they had no problem in conducting these basic physical activities.

In addition to functional abilities, participants emphasised social interaction (“Ability to adapt to the social environment” (+ 3), “State of social relations” (+ 1), “Ability to communicate with people” (+ 1), “social morality” (+ 1)). Participants indicated that people were by nature social beings and cannot live in isolation from society. The importance of social wellbeing was noted by linking it with one’s physical and mental health state. For example, participant 89 mentioned that maintaining good social relations was critical to one’s health by illustrating its positive effects on her health condition: *“If you have some physical or mental health problems, it will be good if you have someone who can listen to you or help you … There was one time when I broke my leg, a lot of my friends came to visit me, cared about me, I felt much better, my bodily pain could even be neglected.” (No.89, female, 56 years old).*

Statements on individuals’ frame of mind, including “Life attitude (such as viewing things optimistically or pessimistically)” (+ 4), “Self-confidence” (+ 3) and “Breadth of mind” (+ 2), were chosen to be most important as well. Participants stated that because it was likely for people to encounter different kinds of troubles or challenges in life, they were supposed to face problems optimistically and confidently to be mentally healthy. Additionally, because a positive thinking frame of mind was believed to be connected with good “ability to adapt to the social environment” and good “state of social relations”, those confident and positive people were more likely to be welcomed by others and more likely to attain social wellbeing. Some participants also mentioned that a positive mental attitude could give one’s body as well as one’s mind a good signal, therefore could positively affect one’s physical and mental states. For example, participant 89 referred to her sister, who stayed positive in fighting cancer and overcame the life-threatening condition as an example to emphasise the positive influence of being optimistic.

Participants in this account did not place much emphasis on psychological feelings: “Feeling of fear” (−5), “Feeling of loneliness” (−3), “Feeling of depression” (−2), “Tendency of being angry” (− 2), “Feeling of anxiety” (− 1) and “Feeling of pressure” (− 1). Some participants said that they normally did not have those negative feelings such as depression or loneliness, therefore, did not think these statements were important in judging health. Some participants stated that they may have experienced some of these feelings but the temporary state of these feelings was “adjustable” and was “not a big deal” (no.37, female, 46 years old). Physical symptoms were also less emphasised in this viewpoint. Statements such as “Feeling of discomfort” (− 2), “Feeling of pain” (− 1), “Feeling of tiredness” (− 1) were ranked as less important, although they were related to physical health. Similar to those psychological feelings, physical symptoms were believed to be short-term in most occasions, therefore could not provide reliable information for health assessment. Some elder participants also explained that these physical feelings were not serious and did not interfere with their normal life. They could still conduct routine activities by tolerating the physical symptoms, thus these symptoms were least important to participants in judging health.

### Factor two: physical health

Q-sorts of 25 participants exemplified this factor.

Physical health is the central focus of this factor. Similar to Factor One, physical function statements including “Ability to wash and dress oneself” (+ 5), “Ability to walk about” (+ 4) “Ability to perform usual activities (such as working, studying, shopping, doing housework)” (+ 3) were ranked as most important. Participants in this group highlighted the importance of being physically independent with comparable reasons reported in the previous factor.

In addition to physical functional abilities, participants highlighted physical symptoms including “Feeling of pain” (+ 4), “Sleep quality” (+ 3), “Feeling of discomfort” (+ 2), “Desire of having food” (+ 2), “Spiritual appearance” (+ 2), “Body strength of doing things” (+ 2) and “Feeling of tiredness” (+ 1) as important health statements. Participants tended to link undesirable physical signs with diseases. If a person got sick, certain physical symptoms such as pain or discomfort would appear in the body, body strength (energy) would be insufficient and he/she may not be able to sleep well or have a good appetite. Meanwhile, it was also believed that if a person did not have good sleep or lost the desire for food, he/she would not have adequate energy and would fall ill easily. Therefore, these physical symptoms could directly reflect one’s health.

Exemplars on this factor showed a clear preference for physical statements. Those statements relating to psychological symptoms as well as cognitive function (such as “Feeling of loneliness”(− 2), “Feeling of fear”(− 2), “Tendency of being angry” (− 1) “Feeling of anxiety”(0), “Feeling of pressure”(0)) as well as cognitive function (such as “Ability to make decisions”(− 4), “Ability to remember things”(− 1), “Ability to concentrate” (− 1) “Ability to think things clearly”(− 1), “Ability to perceive changes in surrounding and to respond”(− 1)) were ranked as less important. Some participants stated that they only considered physical statements to be relevant to health. For example, a participant stated *“Do these (mental) feelings matter? I think a healthy person can also be depressed or fear things… I think if a person can eat and sleep well, he is fine” (no. 55, female, 41 years old)*. Some participants mentioned mental health but emphasised that physical wellbeing was a foundation because mental wellbeing cannot be obtained without a healthy physical body.

In contrast to the first factor where statements relating to social wellbeing were considered most important, participants in Factor Two regarded those statements as least important. According to them, when judging one’s health, it was less important to assess one’s behaviours in front of others (“Social morality” (− 5)) or one’s interaction with other people in the society (“State of social relations” (− 4), “Ability to adapt to the social environment” (− 3), “Breadth of mind” (− 3), “Support from one’s social network” (− 3), “Ability to communicate with people” (− 2)), or one’s personality (“Self-confidence” (− 2)). Some participants considered social wellbeing as “luxuries” (no.81, male, 23). They stated that things like social support or confidence were not necessities for individuals and people could still be healthy even without these things. Some participants said they could not find connections between these statements and health, because, in their understanding, one’s health status was about one’s own condition and was irrelevant to one’s social connections or social environment.

An individual was identified as an exemplar with a Q-sort significantly but negatively loaded on Factor two. This means the individual had a Q-sort that represents a reverse view. For example, he viewed physical function and physical symptom statements as least important, while considered one’s frame of mind as well as social wellbeing as most important. With comparable reasons which were addressed in Factor One, he rated social health and frame of mind highly. He also explained that he did not experience physical functional problems and considered them as least important. The part of the view was shared by Factor Three and Four, which will be presented in the following paragraphs.

### Factor three: sensations and feelings

#### Q-sorts of 16 participants exemplified this factor

The exemplars on this factor were likely to only emphasise health indicators that directly influenced their life. Unlike Factor One and Factor Two where physical functional abilities were placed as most important, participants in this factor did not favour them that much: “Ability to wash and dress oneself” (0), “Ability to perform usual activities (such as working, studying, shopping, doing housework)” (0), “Ability to walk about” (− 1). As a participant explained: *“Walking about, working, dressing myself, I do these things every day. Nothing stops me (from doing these things)… I think people around me, all of them do not have such problems… Only those disabled people have these problems.” (no.53, female, 32 years old).*

For similar reasons, they regarded “Vision” (− 5), “Hearing” (− 4) as well as those cognitive function (“Ability to make decisions” (− 3), “Ability to remember things” (− 3), “Ability to concentrate” (− 2), “Ability to perceive changes in surrounding and to respond” (− 2), “Ability to think things clearly” (− 1)) as less important. It seems that this group of participants did not consider those worst scenarios when people totally lost vision, hearing or cognitive abilities. Some participants illustrated that in his/her age, they were able to see and hear things. They believed that even if people had poor vision or hearing, they could use glasses and hearing-aid and their life would not be affected. Participants also thought it was not likely for people to lose their cognitive abilities until reaching a certain age.

While exemplars viewed physical function as less important in health judgement, they emphasised the importance of physical health by highlighting “Body constitution that can indicate the susceptibility to disease” (+ 5), “Spiritual appearance” (+ 4) and “Sleep quality” (+ 3). Participants generally held the opinion that people with a better body constitution tended to have a lower possibility of developing diseases and were healthier. They also mentioned that spiritual appearance directly indicated one’s health status. Participants described ‘spiritual appearance’ similar to participants in the previous qualitative study. They related this term with an individual’s overall appearance (eye spirit, voice, sitting postures and movement) as well as an individual’s energy. They explained that a person free from diseases and had few things to worry about would generally have a good spiritual appearance, therefore it was a straightforward sign reflecting one’s health.

Participants also tended to regard lifestyle behaviours (“Regularity in daily life” (+ 3), “Diet habits” (+ 2)) as most important. The group held the point that one’s behaviours in daily life would affect or predict one’s health. Good practices such as maintaining a regular life circle, keeping a healthy diet and having a good rest, in this sense, could suggest one’s current health condition and/or could predict one’s future health. Physical feelings including “Feeling of pain” (+ 2), “Feeling of discomfort” (+ 2) and “Feeling of tiredness” (+ 1) were also rated highly by the exemplars of this factor. Most of them explained that they had experienced these undesirable feelings and such feelings had affected their daily life.

Another central theme of this factor was mental wellbeing, as participants tended to highlight the importance of mental health: “Feeling of depression” (+ 3), “Feeling of pressure” (+ 2), “Feeling of anxiety” (+ 2), “Ability to remain stable and peaceful in mood” (+ 1). Exemplars of this group mentioned that people who were mentally unwell may harm themselves, conduct suicide or hurt other people. They thus believed that mental problems were more detrimental than physical diseases. They also tended to agree that nowadays mental problems were more prevalent than physical problems. Additionally, participants held the point that one’s overall health was mainly affected by one’s mental state because those emotions can be controlled subjectively, while one’s physical state tended to be stable and sometimes was not able to be changed. They believed that people could choose to stay in a good mental condition as a way to improve their health, as Participant 71 described: “*Some people are born disabled and it is not fair to say they are unhealthy. They cannot control these objective factors but they can choose to live their own life happily.” (no.71, male, 28 years old)*.

### Factor four: lifestyles

#### Q-sorts of 6 participants exemplified this factor

Similar to Factor Three, exemplars whose sorts defined the fourth factor attached importance to those health dimension indicators, they believed, that had a direct influence on their life. They seemed to be convinced that lifestyles can significantly affect one’s health: “Diet habits” (+ 5), “Regularity in daily life” (+ 4). They tended to believe that people who had regular eating and sleeping time and kept a balanced diet were likely to be better in preventing illness and be healthy, while bad lifestyles undermined one’s health condition. For example, Participants 14 explained:

*“I have a friend who is now 59 (years old) but looks very young. His life is very regular. For most friends of mine, we often play card games until 2 am or 3 am, but he never did that. He would go home by 9 pm… He gets up on time, eats three meals on time, and sleep on time. He swims in the morning.” (no.14, male, 48 years old)*

They also referred to “Sleep quality” (+ 4), “Desire of having food” (+ 3), “Spiritual appearance” (+ 3) and “Body constitution” (+ 3) to be most important health indicators, as they believed those aspects were closely associated with one’s life quality and could straightforwardly reflect one’s health. On the other hand, participants did not emphasise the importance of physical function: “Ability to walk about” (− 4), “Ability to wash and dress oneself” (− 3), “Ability to perform usual activities (such as working, studying, shopping, doing housework)” (− 2), as they believed those things were less likely to affect most people’s normal life because most people would not have problems in these aspects.

While this factor was comparable with Factor Three in terms of the points addressed above, there were distinct differences between the two views. This group of participants recognised cognitive function abilities were important factors in judging health, when they placed “Ability to remember things” (+ 2), “Ability to concentrate” (+ 2), “Ability to make decisions” (+ 1), and “Ability to think things clearly” (0) to be relatively important. This may be because exemplars in Factor Four were older than exemplars in Factor Three, since elder people may not have an as good cognitive function as younger people and/or they may have witnessed more cases where friends/relatives suffered from cognition problems.

Another difference between the Fourth and Third factors was that participants in this group did not regard mental health indicators as most important: “Feeling of fear” (− 5), “Feeling of loneliness” (− 4), “Feeling of pressure” (− 3), “Feeling of depression” (− 3), “Feeling of anxiety” (− 2), “Ability to remain stable and peaceful in mood” (0). This may be because exemplars in this account (similar to Factor One) either had little experience of such negative mental feelings, or they believed such negative feelings could be relieved and would not influence their normal life, as participant 13 explained, *“why people feel anxious or depressed? If some terrible things happen, he would be affected and feel bad. But for most people, if they can sleep well, eat well, have a good body, I think they will hardly feel anxious.” (no.13, male, 50 years old).*

### Factor five: learning and working abilities

#### Q-sorts of 4 participants exemplified this factor

Vision and hearing were the most important health indicators in this factor: “Vision” (+ 5), “Hearing” (+ 4). Participant 68 explained that vision and hearing were essential if a person wanted to be connected with the world and to learn things. If a person lost the ability to see or hear, it became harder for him to get new information (no. 68, male, 24 years old). Yet other physical functional abilities, such as “Ability to wash and dress oneself” (0) and “Ability to perform usual activities” (− 1), were less important to participants because they thought those abilities were too basic for them to worry about.

Participants rated “Frame of mind” highly and chose to believe “Life attitude” (+ 4), “Breadth of mind” (+ 3), “Self-confidence” (+ 2) as most important criteria in judging health. Very similar to the reasons given in Factor one, where frame of mind was regarded as most important, exemplars of Factor Five tended to believe that people who had an optimistic attitude, who were tolerant of things that may be offensive and who were confident were more likely to face challenges and deal with problems in life positively, therefore they were more likely to have a good mental health state. Participants also mentioned the positive influence of a good mental attitude on one’s physical health state.

While exemplars of Factor One considered both frame of mind and social interaction as most important and explained the inner relations between the two, participants in this account did not seem to favour health indicators relating to social wellbeing: “State of social relations” (− 2), “Support from one’s social network” (− 2), “Ability to adapt to the social environment” (− 1), “Ability to communicate with people” (0). This may be explained by the age difference between the two groups of participants. Exemplars in Factor Five (average age 30) were younger than people in Factor One (average age 55). Younger participants may be more concerned about their own work thus did not appreciate social wellbeing as much as exemplars of Factor One.

Participants in this factor placed cognitive abilities, including “Ability to think things clearly” (+ 2), “Ability to concentrate” (+ 2), “Ability to perceive changes in surrounding and to respond” (+ 1), “Ability to remember things” (+ 1), as important statements. They explained that such abilities were vital in their day-to-day work. Similarly, they emphasised “Feeling of pressure”(+ 3) and “Feeling of anxiety”(+ 1) over other mental feelings, because these two feelings were more related to their work, as Participant 20 expressed: *“I cannot control my anxiety. I have too much work stress, even when I go back home, I keep thinking about the work I haven’t finished. I could not sleep, I want to sleep but I can’t.” (no.20, female, 30 years old)* Participant 42 held the point that the statements about anxiety and pressure were more important than “Feeling of depression”, because he had too many responsibilities and stress from work and he had *“no time to be depressed” (no. 42, male, 40 years old)*.

Physical signs or feelings were regarded as less important in this account: “Natural colour and appearance of face” (− 4), “Feeling of pain” (− 3), “Desire of having food” (− 3), “Feeling of discomfort” (− 2), “Feeling of tiredness” (− 2). One reason was that exemplars in this group were relatively young and were less likely to be troubled by negative physical symptoms. Another reason mentioned by participants similar to Factor One: participants tended to believe these physical symptoms were temporary states and could not provide reliable information about an individual’s health status. They believed health was a relatively stable state, except for dramatic changes, such as an accident. Therefore, participants believed that health should not be judged by symptoms that varied from time to time.

## Discussion

### Differences across five factors

The study identified five distinct viewpoints in selecting key indicators that should be used to judge health. Five diverse views in sorting health statements demonstrate that health is a complicated concept and can be understood differently. There were various perspectives in thinking about health: exemplars of Factor One and Two were likely to perceive health from a functional point of view, exemplars of Factor Three tended to define health as the opposite of diseases, while it was widely agreed by exemplars of Factor Four that health was closely linked with one’s lifestyles and daily life quality in terms of sleeping and eating. The five viewpoints also showed a debate between evaluating health as a temporary state or as a longer-term status. While exemplars whose sorts defined Factor One and Five tended to assess health from a long-term basis, participants of Factor Three were likely to perceive health as a short-term state and considered current sensations and feelings as most important. Additionally, findings suggested that diverse priorities were given to different aspects of health in different viewpoints. For example, participants of Factor One jointly highlighted social wellbeing and one’s physical state; Factor Three emphasised mental states; while some exemplars whose sorts generated Factor Two revealed that they considered one’s physical fitness to be most important when thinking about health.

The finding also illustrated how individuals’ demographic characteristics, social surroundings and their own health experiences had shaped their perceptions of health. Similar to the previous literature [42–45], age was found to be one of the most influential factors in shaping lay understandings of health. For example, younger participants were found to talk about mental health more frequently than the elderly. It might be because these young individuals were generally in a better physical health state and were more likely to be exposed to mental health issues. Meanwhile, the elder participants tended to have more physical and cognitive function problems compared to the younger participants and were more likely to highlight the importance of physical and cognitive abilities. In our study, it was also more likely for elder people to raise social wellbeing issues in defining health and this may be because they had more experiences in appreciating the impact of social relations and hoped to be well involved in social communities more [46].

Education was also found to be a salient indicator in shaping participants’ understandings of health, similar to previous findings [43, 44, 47, 48]. While participants with a higher level of education were more likely to be aware of mental health and social wellbeing, participants with a lower level of education were more likely to restrict the scope of health within physical fitness. An extreme example was that several participants who had limited education declared they never heard about “Anxiety” and did not understand its meaning (Participant 27 and 101) and they placed the statement randomly on a less important place. Besides, residence place may also influence individuals’ views according to health. As it was shown in Factor Five, most of the exemplars whose sorts defined this factor lived in cities and illustrated they had a stressful job in a competitive working environment. It may explain why they were more likely to emphasise statements on cognitive abilities and mental health issues.

Apart from demographic characteristics, one’s health conditions and past health experiences influenced one’s interpretation of health [44, 47, 48]. Participants who reported problems in mobility or doing self-care activities were likely to place statements about physical functional abilities as most important. As a result, none of them exemplified Factor Three, Four and Five. It could also be noted that exemplars of Factor Three and Four were generally in a good health state in terms of their EQ-5D results (half of them were in a “11,111” full health state and the majority of them had their self-rating health scores higher than 80). They highlighted the quality of sleeping and eating in judging one’s health and emphasised lifestyle behaviours in maintaining health. It may be because those participants in a better health condition were more likely to think about health in a higher standard and define health in a more positive way. They were less troubled by function limitation or negative feelings/sensations than the participants in the other factors.

### Similarities among the viewpoints

In addition to the differences described above, similarities in understanding the concept of health were also detected across the five factors. There were health dimensions that were important concepts to the majority of participants. The statement “Body constitution that can indicate the susceptibility to diseases” was agreed to be important across the five factors. “Body constitution”, has been closely associated with the concept of health in Chinese populations according to several studies [49, 50]. Its literal translation into Chinese is “body quality” and can be defined as “the characteristics of an individual, including structural and functional characteristics, temperament, ability to adapt to environmental changes, or susceptibility to various health conditions” [51]. This term was found to be an understandable and widely referred concept in describing health among Chinese lay people in published literature [49]. The current Q investigation has further proved it was widely accepted by Chinese participants as an indicator to assess one’s health.

“Spiritual appearance” was another statement that was highly emphasised in the majority of the extracted factors. “Spirit” (“Shen”) is a central notion in traditional Chinese knowledge and could be referred to one’s consciousness, mind, thoughts and/or vitality [52, 53]. A possible description of spirit was illustrated in *Diagnostics in Chinese Medicine* that “having spirit” means “one’s mind is clear, vision is bright, talking is clear, complexion is glowing, facial expression is natural, response is quick, movement is agile, breathing is smooth and steady…” [54] It was recognised in previous qualitative interviews that the concept of “Spirit” was part of lay participants’ common knowledge. Since the statement about spirit was also rated highly in the Q-study, it supported the assumption that “Spirit” could be an important dimension in evaluating health among Chinese communities.

Apart from Factor Two, which firstly prioritised physical health statements, other factors all held the point that one’s “Life attitude (such as viewing things optimistically or pessimistically)” as well as “Breadth of mind” (such as being tolerant of other people or narrow-minded to other people) were most important in judging one’s health. This may reflect Chinese traditional knowledge in appreciating balance and harmony between an individual and the surroundings. It was explained elsewhere that according to Chinese traditional knowledge, because one’s external environment is closely associated with his/her daily activities, ideally, a person should be capable of adjusting to the external environment to reach a harmonious state [55]. It seemed to be widely accepted by many of the participants, as they linked a positive mental-frame with good health and indicated that facing problems in life positively and confidently and avoiding conflicts with other people were good practices in adapting to the changes in the environment to stay in health.

The statement about sleep quality was also extensively agreed to be important, as the four of the extracted factors rated it with a positive importance level and three of them regarded the statement as most important (at least + 3). In a previous scoping review study, sleep was found to be assessed in all identified Chinese-developed HRQoL questionnaires, findings of the current Q study provided additional evidence that sleep was regarded as an important health dimension in China, from an empirical perspective. Life regularity was also rated using positive importance levels by four factors. This may be in line with a phenomenon where the idea of “Yangsheng” (Health-keeping Behaviours) was widely referred to across Chinese communities. The idea conveys that good behaviours, such as keeping a regular lifestyle, can potentially be associated with “good health”. The massive popularity of “Yangsheng” phenomenon in China has been addressed in recent literature [56, 57]. Along with the Q-study result, it indicates that considering one’s behaviours when thinking about one’s health may be common among Chinese lay people.

There were also statements that were agreed to be less important across the five factors. Although adaptability to weather changes was assessed in several Chinese-developed HRQoL questionnaires [58–60], it was not considered to be most important in the extracted views, as most participants revealed that it was less relevant to health compared to other statements. Feelings of fear and loneliness were also found to be less important across the five factors. Participants seemed to agree that their life was not troubled by such two feelings. Some stated they enjoyed their own space and did not regard loneliness was a bad thing. Some stated they did not feel fearful very often thus did not regard it as important. Moreover, “State of sex life” was another statement placed to be less important in the majority of the identified factors. The sensitive nature of this health dimension and its difficulty to be applied in assessing health among Chinese populations was mentioned in the literature [61]. It was also revealed by participants that they concerned this as a private topic and preferred not to discuss it with other people.

### Potential differences in understanding health between China and the west

The findings suggests that Chinese participants’ comprehensions of health were comparable to Western ways of describing health to a great degree. Statements about function abilities, physical symptoms, emotions and social wellbeing were recognised by participants when they were asked to think about health, to various extents though. As those aspects are also principal domains in frequently cited HRQoL conceptual models such as Wilson-Cleary model and PROMIS model [62, 63], it indicates that Chinese and Western HRQoL measures shared comparable measuring frames.

However, it is also clear that there are potential differences in understanding health between China and the West. Comparing the current findings with the descriptive system of EQ-5D, there were unique health dimensions that were agreed to be important among Chinese participants, which are not mentioned in this Western-developed HRQoL questionnaire. Health dimensions including body constitution [49, 50], spirit [55, 64], life attitude [65, 66], sleep [67–69] and life regularity [70, 71] have been frequently linked with the concept of health in the Chinese literature. The importance of such concepts is now supported by the current Q investigation. These “Chinese-characteristic” health dimensions demonstrate cultural differences in defining health between China and the West.

The five dimensions of EQ-5D were included as statements in the study. Although self-care, mobility and usual activities were acknowledged to be important in two of the extracted factors, some participants mentioned that these physical function abilities were too basic for them to worry about therefore were less important. They tended to define health in a more positive way and with a higher standard. Views towards physical feelings of pain and discomfort were also diverse. Some believed they were effective indicators to detect one’s physical health status, while there were participants arguing they could only indicate temporary states and, in most occasions, could not provide reliable information for health assessment. Similarly, anxiety and depression were also believed to be not reliable in evaluating health according to some participants. Furthermore, the two terms may not be well understood by some Chinese people, especially those received limited education.

The results thus imply that the five dimensions of EQ-5D may not be comprehensive in measuring health in China, it can be argued that the questionnaire may fail to ask the most appropriate and important questions among a Chinese population in assessing health.

### Limitations

The study was in nature exploratory. It identified five distinct views of ranking 42 health statements highlighting the most important health statements within each view. However, this method does not provide one overarching or set of statements that were most important to the whole sample of participants. Alternative quantitative research is planned to further investigate the concept of health in China.

Another limitation was that, because the study investigated Chinese lay perceptions of health and recruited only Chinese participants, it was not possible to compare Chinese participants’ views with Westerners’ to explicitly test cultural differences in understanding health between China and the West. A Q-methodological investigation is planned to be conducted in the UK using a similar study design and materials, but potential obstacles in translating the Q-statements into English to make them clear and understandable to Non-Chinese participants are expected.

Although our statements were written, checked for ambiguity and understanding, a small number of participants did not understand some specific statement or interpret some statements differently. For instance, for those statements on functional abilities, some participants imagined situations when one totally lost physical abilities, such as cannot walk or cannot see or cannot hear, thus sorted the statements as most important, while some participants did not expect conditions could be that extreme and did not regard them as most important. We acknowledged that such variations in participants’ interpretation of statements were difficult to control in the sorting exercise and may have influenced how they sorted them.

## Conclusion

Because EQ-5D is one of the most commonly used Western HRQoL measure in China, it was selected as an example to explore cultural differences between China and the West. The Q-study showed that many health statements were rated highly as most important by a diverse range of Chinese participants but were not covered in EQ-5D. It then suggests that the EQ-5D descriptive system might need modification to improve its capacity to measure health status in China. The study raises a general question as to how appropriate the Western-developed HRQoL measures are when used to assess health in a significantly different cultural setting. The findings documented here are specific to China but the implications can and should be considered in other countries/regions.

## Supplementary information

**Additional file 1: Table A1.** the conceptual framework developed from the scoping review and qualitative interviews & how it was transformed into Q-sample.

## Data Availability

The datasets used and/or analysed during the current study are available from the corresponding author on reasonable request.

## Abbreviation

HRQoL: Health-related quality of life

## References

[1] Haraldstad K, Wahl A, Andenæs R, Andersen JR, Andersen MH, Beisland E (2019). A systematic review of quality of life research in medicine and health sciences. Qual Life Res.

[2] Bowling A (2001). Health-related quality of life: conceptual meaning, use and measurement. Measuring disease: a review of disease-specific quality of life measurement scales.

[3] Geisinger KF (1994). Cross-cultural normative assessment: translation and adaptation issues influencing the normative interpretation of assessment instruments. Psychol Assess.

[4] Stewart AL, Napoles-Springer A (2000). Health-related quality-of-life assessments in diverse population groups in the United States. Med Care.

[5] Bowden A, Fox-Rushby JA (2003). A systematic and critical review of the process of translation and adaptation of generic health-related quality of life measures in Africa, Asia, Eastern Europe, the Middle East. South America Soc Sci Med.

[6] Zhou T, Guan H, Liu G, Ma A (2016). Health-related quality of life for disease population in China based on EQ-5D: a systematic review. Zhong guo xun zheng yi xue za zhi.

[7] Zhou T, Guan H, Liu G, Ma A (2016). Health-related quality of life for the general population in China: a systematic review. Zhong guo wei sheng shi ye guan li.

[8] Wang H, Kindig DA, Mullahy J (2005). Variation in Chinese population health related quality of life: results from a EuroQol study in Beijing. China Qual Life Res.

[9] Sun S, Chen J, Johannesson M, Kind P, Xu L, Zhang Y (2011). Population health status in China: EQ-5D results, by age, sex and socio-economic status, from the National Health Services Survey 2008. Qual Life Res.

[10] Tan Z, Liang Y, Liu S, Cao W, Tu H, Guo L (2013). Health-related quality of life as measured with EQ-5D among populations with and without specific chronic conditions: a population-based survey in Shaanxi Province. China PLoS One.

[11] Wu C, Gong Y, Wu J, Zhang S, Yin X, Dong X (2016). Chinese version of the EQ-5D preference weights: applicability in a Chinese general population. PLoS One.

[12] Sun S, Chen J, Kind P, Xu L, Zhang Y, Burström K (2015). Experience-based VAS values for EQ-5D-3L health states in a national general population health survey in China. Qual Life Res.

[13] F-l Z, Yue M, Yang H, Wang T, Wu J-H, Li S-C (2010). Validation and comparison of EuroQol and short form 6D in chronic prostatitis patients. Value Health.

[14] Yang Z, Busschbach J, Liu G, Luo N (2018). EQ-5D-5L norms for the urban Chinese population in China. Health Qual Life Outcomes.

[15] Fang H, Farooq U, Wang D, Yu F, Younus MI, Guo X (2016). Reliability and validity of the EQ-5D-3L for Kashin–Beck disease in China. SpringerPlus..

[16] Wang F, Li H, Ma A (2015). Study on the application of General Utility Scale on Chinese populations. Zhong guo yao wu ping jija.

[17] Wang HM, Patrick DL, Edwards TC, Skalicky AM, Zeng HY, Gu WW (2012). Validation of the EQ-5D in a general population sample in urban China. Qual Life Res.

[18] Zhu Y, Shen Y (2014). A construct validity analysis and application of EQ-5D among type 2 diabetes patients. Zhejiang yu fang yi xue.

[19] Wu C, Gong Y, Wu J, Zhang S, Yin X, Dong X (2016). Chinese version of the EQ-5D preference weights: applicability in a Chinese general population. PLoS One.

[20] Prior L, Chun PL, Huat SB (2000). Beliefs and accounts of illness. Views from two Cantonese-speaking communities in England. Sociol Health Illn.

[21] Liu JE, Mok E, Wong T (2005). Perceptions of Chinese cancer patients of the favorable and unfavorable words conveyed by their social support providers. Cancer Nurs.

[22] Chen AW, Kazanjian A, Wong H (2009). Why do Chinese Canadians not consult mental health services: health status, language or culture?. Transcult Psychiatry.

[23] Xiang YT, Chiu HF, Ungvari GS (2010). Quality of life and mental health in Chinese culture. Curr Opin Psychiatry.

[24] Huang Z, Zhang Y, Ran J, Dong W, Su H, Yang Y (2011). Individual cognition on the concept of health. Zhong guo yun dong yi xue za zhi.

[25] Wang L (2001). A narrative inquiry into the health concept of local adults in Chengdu China.

[26] Watts S, Stenner P (2005). Doing Q methodology: theory, method and interpretation. Qual Res Psychol.

[27] Stephenson W (1935). Correlating persons instead of tests. J Pers.

[28] Stainton Rogers W, Dyson Rogers PO (2001). Q methodological research in mental health and psychotherapy. Qualitative research methods in mental health and psychotherapy: a guide for students and practitioners.

[29] McKeown B, Thomas DB (2013). Q methodology. 2nd ed. Los Angeles; London; New Delhi; Singapore.

[30] Eccleston C, Amanda CDC, Rogers WS (1997). Patients' and professionals' understandings of the causes of chronic pain: blame, responsibility and identity protection. Soc Sci Med.

[31] Stenner P, Dancey C, Watts S (2000). The understanding of their illness amongst people with irritable bowel syndrome: a Q methodological study. Soc Sci Med.

[32] Stenner PH, Cooper D, Skevington SM (2003). Putting the Q into quality of life; the identification of subjective constructions of health-related quality of life using Q methodology. Soc Sci Med.

[33] Peng Y (1998). Democracy and Chinese political discourses. Mod China.

[34] Dewar K, Li WM, Davis C (2007). Photographic images, culture, and perception in tourism advertising: a Q methodology study of Canadian and Chinese university students. J Travel Tour Mark.

[35] Cai D, Stone TE, Petrini MA, McMillan M (2016). An exploration of the health beliefs of Chinese nurses' and nurse academics' health beliefs: a Q-methodology study. Nurs Health Sci.

[36] Bartlett ME, Han W, Bartlett J (2018). Perceptions of mainland Chinese students toward obtaining higher education in the United States. J Int Stud.

[37] Stainton RR, Smith J, Van Langenhove L (1995). Q methodology. Rethinking methods in psychology.

[38] Arksey H, O'Malley L (2005). Scoping studies: towards a methodological framework. Int J Sci Res.

[39] Baker R, Thompson C, Mannion R (2006). Q methodology in health economics. J Health Serv Res Policy.

[40] Dziopa F, Ahern K (2011). A systematic literature review of the applications of Q-technique and its methodology. Eur J Res Meth Behav Soc Sci.

[41] Watts S, Stenner P (2014). Understanding the analytic process (1): factor extraction. Doing Q methodological research: theory, method and interpretation.

[42] Bury M (1982). Chronic illness as biographical disruption. Sociol Health Illn.

[43] Mansour AA-H (1994). The conceptualization of health among residents of Saskatoon. J Community Health.

[44] Baumann B (1961). Diversities in conceptions of health and physical fitness. J Health Hum Behav.

[45] Julia L (2003). Lay experiences of health and illness: past research and future agendas. Sociol Health Illn.

[46] Depp CA, Jeste DV (2006). Definitions and predictors of successful aging: a comprehensive review of larger quantitative studies. Am J Geriatr Psychiatry.

[47] van Dalen H, Williams A, Gudex C (1994). Lay people's evaluations of health: are there variations between different subgroups?. J Epidemiol Community Health.

[48] Blaxter M (2003). Health and lifestyles. London.

[49] Lew-Ting C-Y, Hurwicz M-L, Berkanovic E (1998). Personal constitution and health status among Chinese elderly in Taipei and Los Angeles. Soc Sci Med.

[50] Chan RY, Chien WT (2013). Concepts of body constitution, health and sub-health from traditional Chinese medicine perspective. World J Transl Med.

[51] World Health Organization (2018). International statistical classification of diseases and related health problems (11th Revision).

[52] Rossi E (2007). Shen: psycho-emotional aspects of Chinese medicine.

[53] Zi T (2012). Illustrating the fundamentals of the yellow Emperor’s classic of internal medicine.

[54] Deng T, Guo Z (1984). Diagnostics in Chinese medicine.

[55] Zhang H, Zhang F-Y, Zhao Y-N, Li Z (2015). Health concepts under the view of Chinese culture. Zhong hua hu li za zhi.

[56] Si F, Song X, Gao Y (2013). Current situation of the TCM Yangsheng market. Zhong yi yan jiu.

[57] Sun W (2016). Regimes of healthy living: the reality of ageing in urban China and the cultivation of new normative subjects. J Consum Cult.

[58] Wu D, Lai S, Guo X, Wen Z, Liang W, Yang X (2007). Establishment and initial evaluation of Health Scale of Traditional Chinese Medicine. Zhong guo zhong xi yi jie he za zhi.

[59] Liu FB, Zhao L, Lang JY, Lin LZ, Liang GH, Fang JQ (2007). Development of the Chinese Quality of Life Instrument. Zhong guo zu zhi gong cheng yan jiu he lin chuang kang fu.

[60] Li X (2007). [The development and evaluation of Chinese PRO scale] [master thesis].

[61] Yu C, Sun Y, He L, Bai W, Liu B (2016). Comparative study on the concepts of health related quality of life from TCM and modernized medicine measures. Zhong hua zhong yi yao za zhi.

[62] Wilson IB, Cleary PD (1995). Linking clinical variables with health-related quality of life: a conceptual model of patient outcomes. JAMA..

[63] Cella D, Riley W, Stone A, Rothrock N, Reeve B, Yount S (2010). The patient-reported outcomes measurement information system (PROMIS) developed and tested its first wave of adult self-reported health outcome item banks: 2005–2008. J Clin Epidemiol.

[64] Hsu E (2000). Spirit (shen), styles of knowing, and authority in contemporary Chinese medicine. Cult Med Psychiatry.

[65] An S, Chen C, Li J, Zhang M, Li S, Dou N (2018). Associations of life attitude and personality type with degree of frailty in disabled oldest-old people. Zhong guo gong gong wei sheng.

[66] Li P, Li W (2008). Mentality Health and Physical Health. Jia ting hu shi.

[67] Wang G, Zhang J, Xu Y, Zhu Y, Ke D, Zhang G (2003). Elementary study of sleep quality and self-rated health on common populations. Zhong guo xing wei yi xue ke xue.

[68] Zhao J, Xie Y, Shi M, Ren Z, Feng X (2006). Relationship between sleep quality and quality of life in military personnel in high altitude area. Zhong guo gong gong wei sheng.

[69] Cheng G, Zhang X, Yang Y (2008). Study of Sleep and Quality of Life of the Workers from Chengdu Air Craft Manufacturing Factory. Xian dai yu fang yi xue.

[70] Su D (2015). A study of health beliefs among Chinese adults.

[71] Zhang H, Li H, Gu D (2005). One hundred health proverbs.

