# Supplementary material for: Exploring subjective constructions of health in China: a Q-methodological investigation
Source: Health Qual Life Outcomes. 2020 Jun 3;18:165. doi: 10.1186/s12955-020-01414-z (PMC7268713; doi:10.1186/s12955-020-01414-z)
Supplement: Supplementary file 1 — Additional file 1: Table A1. the conceptual framework developed from the scoping review and qualitative interviews & how it was transformed into Q-sample. [file 12955_2020_1414_MOESM1_ESM.docx]

*Appendix:*

Table A1: the conceptual framework developed from the scoping review and qualitative interviews & how it was transformed into Q-sample.

Top level – Domains: Symptom Status, Function Status

Second Level – Subdomains: Physical Symptom, Psychological Symptom, Physical Function, Cognitive Function, Social Function and Role Function (the Wilson-Clearly model was adopted to form the conceptual framework)

Third Level – Dimensions: sub-components of sub-domains and represent more specific areas. **The statements within the Q-sample were chosen from this level.**

Fourth Level – Items: with even narrower focuses compared to health dimensions. They specifically represent certain aspects for health dimensions.

| **Concepts identified** | | | | **Sources** | | **Q-sample development** | |
| --- | --- | --- | --- | --- | --- | --- | --- |
| **Domain** | **Subdomain** | **Dimension** | **Item** | **Chinese developed HRQoL measures** | **Interviews** | **Q-sample** | **Reasons to exclude** |
| Symptom Status | Physical Symptom | Discomfort | Discomfort in specific body parts | CHPRO, TCMQ, CPSHS, TCMQ | X | Feeling of discomfort |  |
|  |  |  | Discomfort when breathing | CPH42, CPSHS, HSTCM, TCM50, TCMQ |  |  |  |
|  |  |  | Nausea, vomit | CPSHS, TCMQ | X |  |  |
|  |  |  | Palpitation | CPSHS, HSTCM, |  |  |  |
|  |  |  | Dizziness | CPSHS, HSTCM, TCMHSS, TCMQ | X |  |  |
|  |  |  | Dyspepsia | CPSHS, HSTCM, SRHMS | X |  |  |
|  |  |  | Tinnitus | CPSHS, HSTCM, TCMQ |  |  |  |
|  |  |  | Pruritus | TCMQ |  |  |  |
|  |  |  | Discomfort (without specifying) | QOL35, QOLI | X |  |  |
|  |  |  | Numbness | CPSHS | X |  |  |
|  |  | Pain | Pain (without specifying) | CPH42, SRHMS, TCMHSS, TCMQ | X | Feeling of pain |  |
|  |  |  | Painful feelings in specific parts of body | CPSHS, HSQ | X |  |  |
|  |  |  | Intensity of pain | CPH42 | X |  |  |
|  |  |  | Duration of pain | CPH42 | X |  |  |
|  |  | Fatigue | Tiredness | CHPRO, CHQOL, TCM50, TCMHSS, TCMQ, CPH42, HSQ, QOL35, SRHMS | X | Feeling of tiredness |  |
|  |  | Body Strength | Having energy in body to do things | CHPRO, CHQOL, CPSHS, HSTCM, TCM50, TCMHSS, TCMQ, CPH42, QOLI | X | Body strength of doing things |  |
|  |  |  | Willingness to move | CPH42 | X |  |  |
|  |  |  | Breath | CHPRO, CHQOL, TCM50, TCMHSS, TCMQ, CPH42, QOLI |  |  |  |
|  |  | Complexion | Colour of face | CHPRO, CHQOL, TCM50, TCMQ | X | Natural colour and appearance of face |  |
|  |  |  | Colour of lips | CHPRO, CHQOL, TCM50 | X |  |  |
|  |  |  | Eye spirit | CHPRO, CHQOL | X |  |  |
|  |  |  | Shininess on face | CHPRO, CHQOL | X |  |  |
|  |  | Environment adaption | Adaptability to changes in seasons and weather | CHPRO, CHQOL, HSTCM, TCM50 | X | Ability to adapt to weather changes |  |
|  |  |  | Fear of cold/hot weather | HSTCM, TCM50, TCMHSS |  |  |  |
|  |  |  | Adaptability to noisy environment | HSTCM |  |  |  |
|  |  | Body quality | Susceptibility to diseases | TCM50 | X | Body quality that can indicate the susceptibility to diseases |  |
|  |  |  | Inherited body quality |  | X |  |  |
|  |  |  | Susceptibility to catch a cold | HSTCM, TCMHSS | X |  |  |
|  |  | Appearance | Body weight | CPSHS | X | 1) Body weight (is it underweight, normal, overweight or obese) |  |
|  |  |  | Body image | HSTCM, QOLI |  |  |  |
|  |  |  | Body shape |  | X |  |  |
|  |  | Spirit | Spiritual appearance | TCMHSS | X | Spiritual appearance (is it full of spirit or lack of spirit) |  |
|  |  | Appetite | Desire of having food | CHPRO, CHQOL, HSTCM, QOL35, QOLI, SRHMS, TCMHSS | X | Desire of having food |  |
|  |  |  | Food amount | CHPRO, CHQOL, TCMHSS | X |  |  |
|  |  | Other abnormal signs | Stool and urination | CHPRO, HSTCM, TCM50, TCMHSS, TCMQ | X |  | Represented a broad range of sub-items and were too general compared to other health dimensions |
|  |  |  | Feelings in mouth | CHPRO, HSTCM, TCM50, TCMHSS, TCMQ |  |  |  |
|  |  |  | Voice | HSTCM, TCMQ |  |  |  |
|  |  |  | Sweating | CHPRO, HSTCM, TCM50, |  |  |  |
|  |  |  | Skin problems | CPSHS, TCM50, TCMQ |  |  |  |
|  | Psychological Symptoms | Stress | Nervous feeling | CHPRO, CPSHS, HSTCM, QOLI, QOL35, SRHMS | X | Feeling of stress |  |
|  |  |  | Pressure | HSQ, QOLI | X |  |  |
|  |  |  | Stress from work |  | X |  |  |
|  |  |  | Stress from family | CPSHS | X |  |  |
|  |  |  | Economic stress |  | X |  |  |
|  |  | Depression | Sad feeling | CHPRO, CHQOL, CPH42, CPSHS, QOL35, QOLI, SRHMS, TCMHSS | X | Feeling of sadness |  |
|  |  |  | Hopelessness and helplessness | CHPRO, CHQOL, CPSHS, QOLI, SRHMS | X |  |  |
|  |  |  | Tendency to cry | CHPRO, CHQOL, CPSHS | X |  |  |
|  |  |  | Tendency to kill oneself | CPSHS |  |  |  |
|  |  |  | Depression | TCM50 | X |  |  |
|  |  | Anxiety/worry | Worried about things | CHPRO, CHQOL, CPH42, HSTCM, SRHMS, TMCQ | X | Feeling of worry |  |
|  |  |  | Agitated feeling | CHPRO, CPSHS, HSTCM, SRHMS, TCMHSS | X |  |  |
|  |  | Fear | Fear feeling | CHPRO, CHQOL, CPSHS, SRHMS | X | Feeling of fear |  |
|  |  |  | Tendency to be scared | CHPRO, CHQOL, HSTCM, TCM50 |  |  |  |
|  |  | Anger | Tendency to be angry | CHPRO, CHQOL, CPSHS, QOLI, TCM50, TCMHSS | X | Tendency of being angry |  |
|  |  | Emotional stability | Peace in mind | CHPRO, CHQOL, CPH42, QOLI | X | Ability to remain stable and peaceful in mood |  |
|  |  |  | Tendency to be surprised | CPSHS, HSTCM, QOLI | X |  |  |
|  |  |  | Ability to adjust mood |  | X |  |  |
|  |  | Loneliness | Loneliness | CHPRO, CPH42, QOL35, QOLI, SRHMS | X | Feeling of loneliness |  |
|  |  | Confidence | Confidence in oneself | HSTCM, CPH42, CPSHS, QOL35, QOLI, SRHMS |  | Self-confidence |  |
|  |  | Sense of security | Sense of security | CHPRO, CHQOL, CPH42 | X |  | Were opposite to fear and sadness and may create confusions for participants to rank |
|  |  | Happiness | Happiness | CHPRO, CHQOL, CPH42, HSTCM, QOL35, QOLI, SRHMS, TCM50 | X |  |  |
|  |  | Satisfaction | Satisfaction with life | CHPRO, CPH42, HSTCM. SRHMS |  | Sense of satisfaction with life |  |
| Functional Status | Physical Function | Sleep | Insomnia | CHPRO, CHQOL, CPSHS, HSTCM, TCM50, TCMHSS, TCMQ | X | Sleep quality |  |
|  |  |  | Sleep quality | CHPRO, CHQOL, HSQ, HSTCM, QOLI, SRHMS, TCMHSS, TCMQ | X |  |  |
|  |  |  | Sleep length | CPH42 | X |  |  |
|  |  | Mobility | Ability to walk about | CPH42, QOL35, QOLI, SRHMS | X | Ability to walk about |  |
|  |  |  | Ability to take stairs | CPH42, QOL35, SRHMS | X |  |  |
|  |  |  | Ability to bend ones’ knees | CPH42, QOL35, SRHMS | X |  |  |
|  |  |  | Flexibility |  | X |  |  |
|  |  |  | Ability to run/exercise | QOL35, SRHMS | X |  |  |
|  |  | Usual activities | Ability to go shopping | QOL35, QOLI, SRHMS | X | Ability to perform usual activities (such as working, studying, shopping, doing housework) |  |
|  |  |  | Ability to do housework | QOL35, QOLI, SRHMS | X |  |  |
|  |  |  | Ability to feed oneself | SRHMS | X |  |  |
|  |  | Self-care | Ability to dress, bath oneself | CPH42, QOL35, QOLI, SRHMS | X | Ability to take care of oneself (such as washing and dressing oneself) |  |
|  |  | Organ function | Vision | CPSHS, QOLI, SRHMS, TCMQ | X | 1) Vision  2) Hearing |  |
|  |  |  | Hearing | CPSHS, QOLI, SRHMS, TCMQ | X |  |  |
|  |  |  | State of teeth and gums | TCMQ |  |  |  |
|  |  |  | State of chest and abdomen | TCMQ |  |  |  |
|  |  |  | State of heart |  | X |  |  |
|  |  |  | State of limbs | TCMQ |  |  |  |
|  |  | Communication | Ability to communicate with people | CPSHS, HSTCM, TCM50 | X | Ability to communicate with people |  |
|  |  |  | Ability to speak clearly | CHPRO, CHQOL | X |  |  |
|  |  |  | Ability to express ideas clearly | CHPRO, CHQOL |  |  |  |
|  |  | Dependence on medicine | Dependence on medication | QOL35, QOLI |  | Dependence on medication |  |
|  |  | Sexual function | Satisfaction with sex life | CPSHS, QOL35 |  | State of sex life |  |
|  |  |  | State of sex life | QOLI |  |  |  |
|  | Cognitive Function | Memory | State of memory | CHPRO, CHQOL, TCM50, TCMHSS, TCMQ, QOL35, SRHMS | X | Ability to remember things |  |
|  |  |  | Ability to remember things | QOLI | X |  |  |
|  |  | Thinking | Ability to think clearly | CHPRO, CHQOL, CPH42, HSTCM, QOLI, SRHMS | X | Ability to think things clearly |  |
|  |  |  | Being clear-headed or in confusion | CHPRO, CHQOL, CPH42 | X |  |  |
|  |  | Reaction | Ability of to perceive changes in surrounding and to respond swiftly | CHQOL, QOLI | X | Ability of to perceive changes in surrounding and to respond swiftly |  |
|  |  |  | Speed of response | CHPRO, CHQOL | X |  |  |
|  |  | Concentration | Ability to concentrate | CHPRO, CHQOL, CPH42, HSTCM, QOL35, QOLI, SRHMS, TCM50 | X | Ability to concentrate |  |
|  |  | Decision-making | Ability to make decisions | CPSHS, HSTCM, QOLI | X | Ability to make decisions |  |
|  | Social/Role Function | Role activities | Ability to conduct usual role activities (work role, family role, study role) | CHPRO, QOLI, SRHMS, TCMQ | X |  | Included in “Ability to perform usual activities” |
|  |  | Social relations | Satisfaction with social relations including relations with friends, family and colleagues | QOLI | X | State of social relations (such as the relations with family, friends or colleagues) | Too similar to social relation according to pilot study |
|  |  |  | Quality (good or bad) of social relations including relations with friends, family and colleagues | CHPRO, CPH42, CPSHS, QOL35, QOLI, SRHMS | X |  |  |
|  |  |  | Quantity of social relations (enough friends) | CPH42, SRHMS | X |  |  |
|  |  | Social support | Satisfaction with the support from family and friends | CHPRO, QOLI, SRHMS | X | State of the support from one’s social network (such as supportive resources from friends and family. |  |
|  |  |  | Existence of social support | CPH42, QOL35 | X |  |  |
|  |  |  | Ability and willingness to offer support | QOL35, QOLI, SRHMS |  |  |  |
|  |  | Social adaption | Ability to adapt to social environment, to adapt to policies/regulation | CPH42, CPSHS, HSQ, SRHMS | X | Ability to adapt to the social environment (such as working environment, living environment, social rules and regulations) |  |
|  |  | Morality | One’s s willingness to follow moral norms. | CPSHS | X | Social morality (does he/she follow moral norms) |  |
|  |  | Social contact | Frequency of participating in communal activities or contacting with relatives and friends | QOLI, SRHMS |  |  |  |
| Other | Other | Life attitude | One’s attitude towards life (e.g. viewing things optimistically or pessimistically). | HSTCM, QOLI, SRHMS | X | Life attitude towards life (such as viewing things optimistically or pessimistically) |  |
|  |  | “Breadth of mind” | One’s attitude to other people. (e.g. being tolerant of other people or narrow-minded) |  | X | “Breadth of mind” (such as being tolerant of other people or narrow-minded to other people) |  |
|  |  | Personality | Character flaws, problems in personality |  | X |  | Represented a broad range of sub-items and were too general compared to other health dimensions |
|  |  |  | Positive personality including being friendly, generous, open-minded and brave. |  | X |  |  |
|  |  | Lifestyle | Diet habits | CPSHS, HSQ | X | 1) Diet habits  2) Regularity in daily life |  |
|  |  |  | Regularity in daily life | HSQ | X |  |  |
|  |  | Family medical history | Family medical history | CPSHS | X | Family medical history (Whether his/her close relatives diagnosed with critical illnesses) |  |
|  |  | Overall health rating | Overall health status | HSTCM, QOL35, QOLI, SRHMS |  |  | Too general |
|  |  | Overall QOL | Overall quality of life | CHPRO, QOLI, QOL35 |  |  |  |
|  |  | Economic status | Economic status | CHPRO, QOL35, QOLI |  |  | Not included as health dimensions defined here |
|  |  | Living environment | Living environment | QOL35, QOLI, SRHMS |  |  | Not included as health dimensions defined here |
|  |  | Disease | Disease |  | X |  | Too general |
|  |  | Physical examination results | Physical examination results |  | X |  | Too general |

The abbreviations for the Chinese HRQoL measures:

TCMHSS, 中医健康状况量表, TCM-HSS, Liu et al, 2008

CHPRO, 中华PRO量表, CHPRO, Li, 2007

HSTCM, 中医健康量表, Health Scale of TCM, Wu and Lai, 2007

CHQOL中华生存质量量表, Chinese QoL Instrument, Liu et al, 2007

HSQ, 健康状况测评问卷, Health Status Questionnaire, Tian et al, 2009

TCM50, 中医健康状态自评问卷(中医自测量表-50), Self-Rating Questionnaire of Health Status in Traditional Chinese Medicine, Wang et al, 2011; Zhang et al, 2017

TCMQ, 中医健康状态测试, Questionnaire (the First Edition) Based on TCM for Detecting Health Status, Zhou et al, 2015

QOL35, 国人生活质量普适量表, QOL35, Wu et al, 2005

SRHMS, 自测健康评定量表, Self-rated Health Measurement Scale, Xu et al, 2000

CPH42, 自感健康量表, Current Perceived Health Questionnaire-42, Fielding and Li, 1997

QOLI, 生活质量综合评定问卷 , Quality of Life Inventory, Li et al, 1995

CPSHS, 中国心身健康量表Chinese Psychosomatic Health Scale, Zhang et al, 1993

**Q-sample:**

评判一个人的健康状况时，了解这个人的____有多重要？

When judging a person’s health, how important is it to know about their ________?

|  | Chinese statements | English language equivalent | Source^[[1]](#footnote-1)^ |
| --- | --- | --- | --- |
| 1 | 身体素质所对应的对疾病的抵抗力 | Body quality that can indicate the susceptibility to diseases | SR&Qua |
| 2 | 对天气变化的适应能力 | Ability to adapt to weather changes | SR&Qua |
| 3 | 身体体重 （是偏瘦，正常，偏重还是肥胖） | Body weight (whether he/she is underweight, normal, overweight or obese) | SR&Qua |
| 4 | 精神面貌 （是精神饱满还是无精打采） | Spiritual appearance (whether he/she is full of spirit or lack of energy) | Qua |
| 5 | 面色 | Natural colour and appearance of face | SR&Qua |
| 6 | 疲劳、困倦想睡觉的感受 | Feeling of tiredness | SR&Qua |
| 7 | 体力，身体四肢的力气 | Body strength of doing things | SR&Qua |
| 8 | 身体不舒服的感受 （例如头晕，恶心，心悸等） | Feeling of discomfort (such as dizziness, nausea, palpitation) | SR&Qua |
| 9 | 身体上疼痛的感受 | Feeling of pain | SR&Qua |
| 10 | 胃口，食欲 | Desire of having food | SR&Qua |
| 11 | 精神的压力与紧张 | Feeling of pressure | SR&Qua |
| 12 | 抑郁、心情低落的心理状态 | Feeling of sadness | SR&Qua |
| 13 | 焦虑不安的心理状态 | Feeling of being worried | SR&Qua |
| 14 | 容易生气发火的性情 | Tendency of being angry | SR&Qua |
| 15 | 恐惧感 | Feeling of fear | SR&Qua |
| 16 | 孤独感 | Feeling of loneliness | SR |
| 17 | 自信心 | Self-confidence | SR |
| 18 | 调整心情，使心情平和的能力 | Ability to remain stable and peaceful in mood | SR&Qua |
| 19 | 睡眠的质量 | Sleep quality | SR&Qua |
| 20 | 四处走动的能力 | Ability to walk about | SR&Qua |
| 21 | 进行日常活动的能力（例如上学工作，上街，做家务事） | Ability to perform usual activities (such as working, studying, shopping, doing housework) | SR&Qua |
| 22 | 视力 | Vision | SR&Qua |
| 23 | 听力 | Hearing | SR&Qua |
| 24 | 交流沟通的能力 | Ability to communicate with people | SR&Qua |
| 25 | 自理的能力（如自己给自己穿衣、洗澡） | Ability to wash and dress oneself | SR&Qua |
| 26 | 日常生活对药物的依赖程度 | Dependence on medication | SR |
| 27 | 性生活的情况 | State of sex life | SR |
| 28 | 清楚思考的能力，大脑清晰度 | Ability to think things clearly | SR&Qua |
| 29 | 反应力，对外部环境的变化敏捷作出反应的能力 | Ability of to perceive changes in surrounding and to respond swiftly | SR&Qua |
| 30 | 记忆力，能记忆事物的能力 | Ability to remember things | SR&Qua |
| 31 | 决策力，需要做选择时的决断程度 | Ability to make decisions | SR&Qua |
| 32 | 注意力，集中精神的能力 | Ability to concentrate | SR&Qua |
| 33 | 人际关系的情况（家庭关系、与朋友的关系，与同事的关系等） | State of social relations (such as the relations with family, friends or colleagues) | SR&Qua |
| 34 | 融入社会环境的能力（如适应工作、生活环境，适应社会法律制度） | Ability to adapt to the social environment (such as working or living environment, social rules and regulations) | SR&Qua |
| 35 | 获得的社会支持的情况（如家人、朋友的支持） | State of the support from one’s social network (such as supportive resources from friends and family. | SR&Qua |
| 36 | 公德心（行为是否遵从社会公德） | Social morality (whether he/she follow moral norms) | SR&Qua |
| 37 | 人生态度（比如对待生活是否积极乐观，做事积极或是消极） | Life attitude towards life (such as viewing things optimistically or pessimistically) | Qua |
| 38 | 心胸（比如心胸宽广或是狭隘，是否对他人经常抱怨、耿耿于怀） | “Breadth of mind” (such as being tolerant of other people or narrow-minded to other people) | Qua |
| 39 | 生活作息的规律性 | Regularity in daily life | SR&Qua |
| 40 | 饮食习惯 | Diet habits | SR&Qua |
| 41 | 生活的满足感 | Sense of satisfaction with life | SR |
| 42 | 家族疾病史 （近亲属是否患有重大疾病） | Family medical history (whether his/her close relatives diagnosed with critical illnesses) | SR&Qua |

**Examples of post-sorting questions:**

1) Why did you choose ___ as the most important statements? Why did you choose ___ as the least important statements? How did you understand those health statements that were chosen to be the most/least important?

2) When we are judging one’s health, do you think there are additional important issues that are missing from these statements? 3) Are there any statements that you did not understand or you would like to comment on?

1. The sources of Q-sample were from either the scoping review study or the qualitative interviews. In the table, “SR” stands for the scoping review study, “Qua” represents the qualitative interview study. [↑](#footnote-ref-1)
